# Chloroform in Infantile Convulsions

**Published:** 1852-05

**Authors:** 


*Chloroform in Infantile Convulsions.*—This remedy is highly recommended in the convulsions of infants, by Dr. Simpson, of Edinburgh, in cases where they depend upon reflex, eccentric, or sympathetic causes.

